# Editorial: Multi-omics strategy toward identifying and managing hereditary and metabolic liver diseases

**DOI:** 10.3389/fgene.2024.1520721

**Published:** 2024-11-27

**Authors:** Mingjie Wang, Sujun Zheng, Xinhua Li

**Affiliations:** ^1^ Department of Gastroenterology, Ruijin Hospital, Shanghai Jiao Tong University, School of Medicine, Shanghai, China; ^2^ First Department of Liver Disease, Beijing YouAn hospital, Capital Medical University, Beijing, China; ^3^ Department of Infectious Diseases, The Third Affiliated Hospital, Sun Yat-sen University, Guangzhou, China

**Keywords:** multi-omics, hereditary liver disease, metabolic dysfunction associated steatotic liver disease, machine learning, big data

## Introduction

Liver diseases encompass a broad and complex medical domain, representing a significant global health challenge with serious implications for affected individuals and healthcare systems worldwide. Among these, hereditary and metabolic liver diseases pose distinctive challenges due to their intricate genetic and metabolic underpinnings. Recently, the landscape of bio-medical research has recently been revolutionized by the growing field of omics technology which includes genomics, transcriptomics, metabolomics, proteomics and epigenomics. These cutting-edge technologies provide unrivalled opportunities that help researchers explore biological systems at proportions that were previously unimagined, hence leading to a more subtle understanding of disease mechanisms. In the present Research Topic, several studies have shown that integration of different datasets from various omics is set to propel the management of hereditary and metabolic liver diseases through identification of novel biomarkers, unraveling complex biological pathways or revealing new therapeutic avenues.

### Genomic advances in liver disease research

Genomic technologies have been pivotal in elucidating the genetic basis of hereditary liver diseases. Historically, clinicians and researchers have a complicated architecture to resolve such issues on Wilson’s disease, hemochromatosis and alpha-1-antitrypsin deficiency, and so on. Recent advancements in genomic sequencing techniques have facilitated more precise and rapid identification of pathogenic mutations, largely due to high-throughput sequencing methods such as targeted gene panel sequencing and whole-exome sequencing (WES). For example, Ronzoni et al. provided a targeted sequencing of 82 genes associated with chronic liver diseases, and advocated targeted gene panel sequencing technique that would aid in avoiding deleterious mutations. Their refined method exhibited a higher mean depth of coverage within target regions and similar specificity to WES, highlighting the value of targeted genomic analyses. This type of focused approach increases cost efficiency as well as diagnostic yield in cases that require high rapid turnaround time.

### Integrative pan-cancer analysis applied to liver diseases

The integration of pan-cancer analyses as reported by Zeng et al. in their exploration of the G6PD gene, optimistically opens a new avenue in terms of interrogating liver diseases. G6PD, a pivotal enzyme in the pentose phosphate pathway, emerges as a prognostic marker and therapeutic target across various cancers, including liver cancer. Liver cancer, often a terminal stage in the progression of certain liver diseases, benefits from insights gained through cancer-wide analyses that reveal common molecular drivers. Understanding the role of such genes can uncover shared pathological pathways and offer new strategies for intervention and treatment tailored specifically to liver cancer. For example, the dysregulation of G6PD and its interactions with other metabolites could affect cell proliferation, apoptosis, and cellular stress responses, underscoring its relevance in both metabolic and liver cancer pathologies.

### Integration of omics datasets: a comprehensive perspective

A systemic participation of the various omics datasets is fundamental for the comprehension of hereditary as well as metabolic liver diseases. Ye et al. highlight this approach through their analysis of non-alcoholic fatty liver disease (NAFLD), employing extensive transcriptome datasets to reveal crucial gene co-expression modules. By integrating multi-dimensional data, it is possible to address complex relationships among genotypic, transcriptomic, and phenotypic variation. Such integrative efforts expedite the search for new potential drug targets and biomarker discoveries. In particular, the genetic modules that are implicated in the development and progression of NAFLD, are important for the diagnosis and targeting therapy, which is a positive move towards precision medicine and targeted healthcare.

### The role of artificial intelligence in liver disease research

Nowadays, machine learning and artificial intelligence (AI) have made their way to the forefront of biomedical research. Its accuracy and efficiency in processing information across large datasets makes it invaluable for interpreting multi-omics data in liver disease research. The study by Yu et al. illustrates the successful implementation of machine learning algorithms to discern predictive biomarkers in molecular clusters associated with disulfidptosis in NAFLD. Among their sophisticated models used, support vector machine (SVM) stands out having demonstrated sufficient discriminatory power, revealing its potential for precise disease prediction and management. The incorporation of AI into multi-omics approaches assists in the creation of an integrated database for disease diagnosis, prognosis, and therapy selection. Furthermore, AI-driven models offer predictive insights into disease course based on patient-specific profiles, paving the way for highly personalized treatment plans.

### Challenges and opportunities in multi-omics strategies

The multi-omics approaches exhibit their vast potential, yet still a few challenges remain. The first major obstacle is the effective construction of multi-dimensional datasets. There is a huge level of complexity across each of the omics layers such as genomics or epigenomics that require huge amounts of bioinformatics tools and integrative algorithms for the integration to make sense. Effective data normalization and standardization remain pivotal to ensure cross-study comparisons and accurate result interpretations. Absence of such processing will allow variation in experimental conditions or sample, and finally lead to spurious results. In addition, bioinformatics tools have to be improved continuously for the increasing complexity and volume of data. The translational challenge from bench to bedside involves validating omics-derived biomarkers and therapeutic targets through rigorously designed clinical trials. Ensuring that *in vitro* and *in silico* findings translate into clinical efficacy is no simple feat, requiring careful consideration of diverse patient populations and disease heterogeneity. Furthermore, the use of new diagnostics and treatments must be guided by ethical as well as legal considerations.

## Conclusion

In conclusion, although hurdles remain, the promise of a multi-omics strategies in transforming the pursuit of solutions to hereditary and metabolic liver diseases is undeniable. With the help of umpteenth integration of different omics datasets along with AI, it is expected that new insights into these complex disease conditions will be found which will revolutionize the way of research. Further investigation and thoughtful combination of the technologies developed into clinical practice will be essential in order to develop more effective and personalized treatments which will significantly maximize patient benefits.

